# PMMA-augmentation of the spinous process as an enhancing-protective measure against bone failure in “through the spinous process-vertebropexy”

**DOI:** 10.1016/j.xnsj.2025.100842

**Published:** 2025-12-20

**Authors:** Alexandros Tsolakidis, Marie-Rosa Fasser, Oliver Wigger, Mazda Farshad, Jonas Widmer

**Affiliations:** a Department of Orthopedics, Balgrist University Hospital, University of Zurich, Forchstrasse 340, 8008 Zurich, Switzerland; b University Spine Center Zurich, Balgrist University Hospital, University of Zurich, Zurich, Forchstrasse 340, 8008 Zurich, Switzerland; c Spine Biomechanics, Department of Orthopedic Surgery, Balgrist University Hospital, University of Zurich, Forchstrasse 340, 8008 Zurich, Switzerland; d Institute of Biomechanics, ETH Zurich, Balgrist Campus, Lengghalde 5, 8008 Zürich, Switzerland

**Keywords:** Allograft, Fusion, Ligament, Lumbar spine, Spinal stabilization, Spinous process, Vertebropexy, Xenograft, PMMA, Cement-augmentation

## Abstract

**Background:**

Vertebropexy, a semi-rigid spinal stabilization technique, utilizes the spinous process(SP) as an anchor point for stabilizing tendon-grafts or flexible cerclages. In its primary form, it entailed drilling into the bone of 2 adjacent SPs and threading the materials through the holes. Biomechanical studies have identified the SP as the weakest part of the vertebrae, while cadaveric studies have demonstrated a higher bone failure rate with osteoporosis. We investigated whether cement augmentation of the SP could enhance the biomechanical strength and reduce the fracture-risk in the setting of first-generation Vertebropexy.

**Methods:**

Following computed tomographic analysis and measurement of the bone mineral density, 12 lumbar segments were divided in 2 groups (Osteoporotic/ Nonosteoporotic) and then fixed in custom-made 3D-printed clamps. The SPs of 6 segments underwent cement augmentation (PMMA-Group), and a CT scan confirmed adequate augmentation. The other 6 segments remained uncemented. (Native-Group). Posterior decompression, drilling, and instrumentation with bovine tendons were then conducted. Torque-to-failure stress tests were performed on a biaxial static testing machine.

**Results:**

The cement-augmentation of the SP significantly increases the torque-to-failure in flexion (p=.00037/ Median & IQR: 13.0 & 5.2 Nm in the Native-Group vs. 26.5 & 11.1 Nm in the PMMA-Group), regardless of the bone quality (p=.008). A statistically significant difference in torque-to-failure between Osteoporotic and NonOsteoporotic groups inside the PMMA and Native groups was determined (p=.015 and p=.025, respectively). A statistically significant correlation between bone density and failure torque was not detected in this cohort, possibly due to the limited sample size (Spearman 0.276, p=.192). A comparison between the torque-to-failure of the Native-NonOsteoporotic SPs and that of the PMMA-Osteoporotic showed no statistical significance (p=.240).

**Conclusions:**

Based on the findings of this small-sample cadaveric study, cement-augmentation of the spinous processes can multiply the torque-to-failure/fracture in both osteoporotic and nonosteoporotic conditions and may be used as a salvage technique in first-generation vertebropexy procedures that compromise the spinous process.

## Background

Posterior spinal fusion is one of the most common therapeutic approaches for various spinal disorders, consistently delivering reliable short-term outcomes [1]. However, complications related to implants, bone healing, and neurovascular injury, as well as issues such as adjacent segment disease and postural changes [[2], [3], [4], [5]], result in approximately one-third of patients requiring revision surgery within 15 years [6]. Although alternative techniques for rigid or semi-rigid spinal stabilization or soft-landing techniques to prevent degeneration of the adjacent segments [7,8] have been developed, they have not demonstrated superior long-term outcomes compared to spinal fusion. These alternatives often lead to new complications at the implant-bone interface, such as device fracture, dislocation, and screw loosening, and typically involve complex surgical procedures with a steep learning curve [[9], [10], [11], [12], [13]]. Additionally, these techniques are associated with high reoperation rates and low cost-effectiveness [11,13]. Consequently, their use in clinical practice remains limited.

**Fig. 1:**
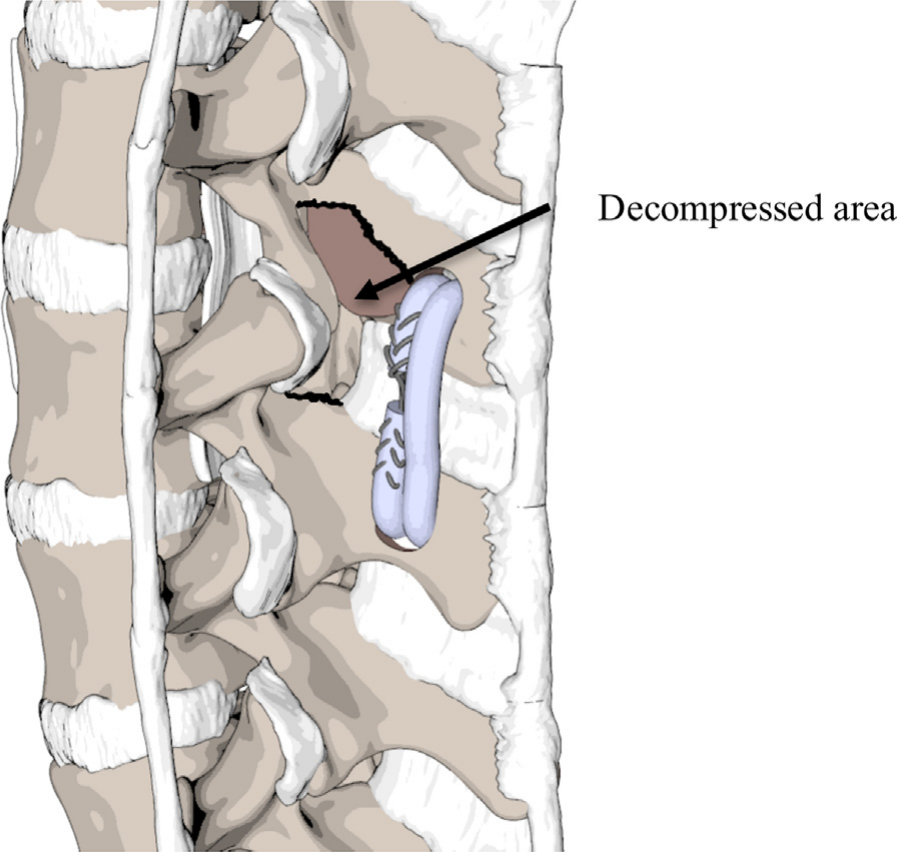
The first generation of VPX, which was used in our experiments.

To address the complexities and complications associated with traditional spinal fusion, a new therapeutic approach called "Vertebropexy" (VPX) was developed [9] [Fig. 1]. This technique involves ligamentous reinforcement of the vertebrae, stabilizing the spine by inserting tendinous grafts to counteract excessive painful motion and spinal instability [10] without completely immobilizing the segment. The primary objective is to provide relative stabilization of the targeted segment, particularly in clinically significant directions of motion such as flexion and shear movements. This stabilizing procedure has demonstrated promising biomechanical outcomes [9] and short-term clinical results without the complications typically associated with spinal fusion [14].

However, new challenges have emerged due to the anatomical and biomechanical properties of the bony components involved in VPX and the technique itself. The first-generation VPX procedure required drilling into the bone of 2 spinous process structures (SP) that may already be weakened by decompression [15] and threading stabilizing materials through the drill holes. Biomechanical studies have identified the SP as the weakest part of the vertebra, and several cadaveric studies have shown a significantly higher, or trending towards higher, rate of bone failure in the SP under osteoporotic conditions [16,17]. Furthermore, SP fractures are a clinically observed complication following first-generation (through the SP) VPX. The torque, especially during flexion, can play a significant role in the failure of the decompressed spinal segments undergoing VPX [18].

We hypothesize that to mitigate these issues in first-generartion through the SP VPX, a minimally invasive PMMA augmentation of the cancellous bone in the SP, performed before drilling/ anchoring, could potentially enhance the biomechanical strength of the bone and reduce the risk of complications.

## Materials and methods

### Dissection, preparation and storage

After obtaining approval from the hospital's investigational board and the cantonal Ethic Committee of Zurich, twelve lumbar spinal segments (TH12/L1: 4, L2/3: 4, L4/5: 4) were extracted from 4 fresh-frozen cadavers (Table 1; Science Care, Phoenix, AZ, USA). Computed tomography (CT, Naeotom Alpha, Siemens Healthineers, Erlangen, Germany) scans of the donor tissue were obtained, allowing the samples to be divided into 2 groups of equivalent size: 1 with normal bone quality or osteopenia (Nonosteoporotic) and another containing osteoporotic specimens (Osteoporotic). It was further ensured that no bony lesions were present. Categorisation of bone quality was done based on bone mineral density (BMD) of the vertebral body spongiosa: Osteoporosis BMD<80mg/cm3, osteopenia 80<BMD<120 mg/cm3 and normal BMD>120 mg/cm3 [19]. Tissue BMD was determined using the QRM Bone Density Calibration Phantom 6 H700 [20]. The measurements of the density of the trabecular bone of the vertebral body in mean Hounsfield Units were done within a circular area with a minimum diameter of 5 mm on axial cuts. Based on the corresponding density of the Phantom, the BMD was calculated.

**Table 1:** The characteristics of the donors.

Name	Weight (kg)	Height (m)	Age (y)	Sex assigned at birth	Comorbidities	Vertebra	Bone density of vertebral body (mg/cm3)	Bone density of spinous process (mg/cm3)	PMMA augmentation
1	62	1.57	83	Female	Alzheimer's disease	T12	70.40	30.60	Yes
						L1	72.65	54.00	Yes
						L2	78.90	77.80	No
						L3	65.94	58.55	No
						L4	70.98	78.97	Yes
						L5	75.12	71.05	Yes
2	57	1.45	66	Female	Atherosclerotic heart disease, tobacco, obesity	T12	127.43	124.76	No
						L1	128.73	133.00	No
						L2	131.31	130.35	Yes
						L3	142.79	180.74	Yes
						L4	158.30	175.12	No
						L5	133.27	215.11	No
3	70	1.78	69	Female	COPD	T12	59.83	59.92	No
						L1	63.57	39.74	No
						L2	67.54	60.79	Yes
						L3	35.44	37.58	Yes
						L4	52.26	47.62	No
						L5	63.98	64.69	No
4	67	1.70	62	Female	Metastatic gastric cancer	T12	123.81	163.30	Yes
						L1	112.10	116.56	Yes
						L2	86.66	115.81	No
						L3	89.44	87.82	No
						L4	95.30	128.21	Yes
						L5	119.68	124.94	Yes

Abbreviations: m, months; y, years.

The spinal segments were dissected carefully to preserve the bony processes and the intrasegmental paraspinal ligaments, facet joint capsules, and the intervertebral disc.

### Group design

Spinal segments were randomly assigned to the Native or PMMA group using a random number generator. No stratification was performed to ensure a balanced distribution of osteoporotic and nonosteoporotic specimens between the PMMA and Native groups. However, there was ultimately no significant difference in bone mineral density (BMD) between groups (92.4±35.4 vs. 93.1±32.6 mg/cm³). When classified by bone quality, a significant difference in BMD was observed: Normal bone (121.0±21.6 mg/cm³) vs. Osteoporotic bone (64.7±11.6 mg/cm³).

### Microsurgical decompression and PMMA augmentation

The remnants of the adjacent supraspinous and interspinous ligaments and the adjacent facet joint capsules were sharply removed using a Leksell rongeur. The intrasegmental posterior longitudinal ligament (PLL), facet joint capsule (FJC), ligamentum flavum (LF), interspinous ligament (ISL), and supraspinous ligament (SSL) were left intact.

A standard vertebroplasty needle set (Depuy Synthes) and standard PMMA bone cement (Vertacem *V* + -Depuy) were employed for PMMA augmentation of the SP. After drilling with a 1.6 mm K-wire, which served as a guide, a 10-G vertebroplasty working sleeve with a cannulated trocar was inserted into the posterior edge of each SP using a posterior midline approach without removing the SSL. The sleeve/trocar system was carefully advanced, rotating it manually along the center and mid-distance of the SP from the caudocranial height, penetrating approximately 0.5 cm without a hammer. Once the working sleeve was stabilized, the cannulated trocar and K-wire guide were removed, and an access drill was used to prepare the medullary bone up to the spinolaminar junction, avoiding breaching the ventral cortex of the lamina. The access drill was removed, and a PMMA needle with a side opening was advanced to the spinolaminar junction to ensure its PMMA augmentation as well as that of the anterior SP.

Careful, slow injection of regular vertebroplasty PMMA with very low viscosity was performed using 2 mL syringes. The PMMA components were cooled for 24 hours at 5°C before use to prolong the polymerization phase and maintain PMMA fluidity as long as possible. 4 mL of PMMA was injected while the needle was repositioned, rotating clockwise and gradually withdrawn to augment the SP's dorsal parts. Extraosseous leakage, observed through visual inspection and CT imaging, was minimal, with only minor venous leaks from the lateral edges of the SP and minimal ventral leaks, all contained posterior to the LF. Posterior leakage was controlled by applying minimal pressure with the surgeon's fingers.

**Fig. 2:**
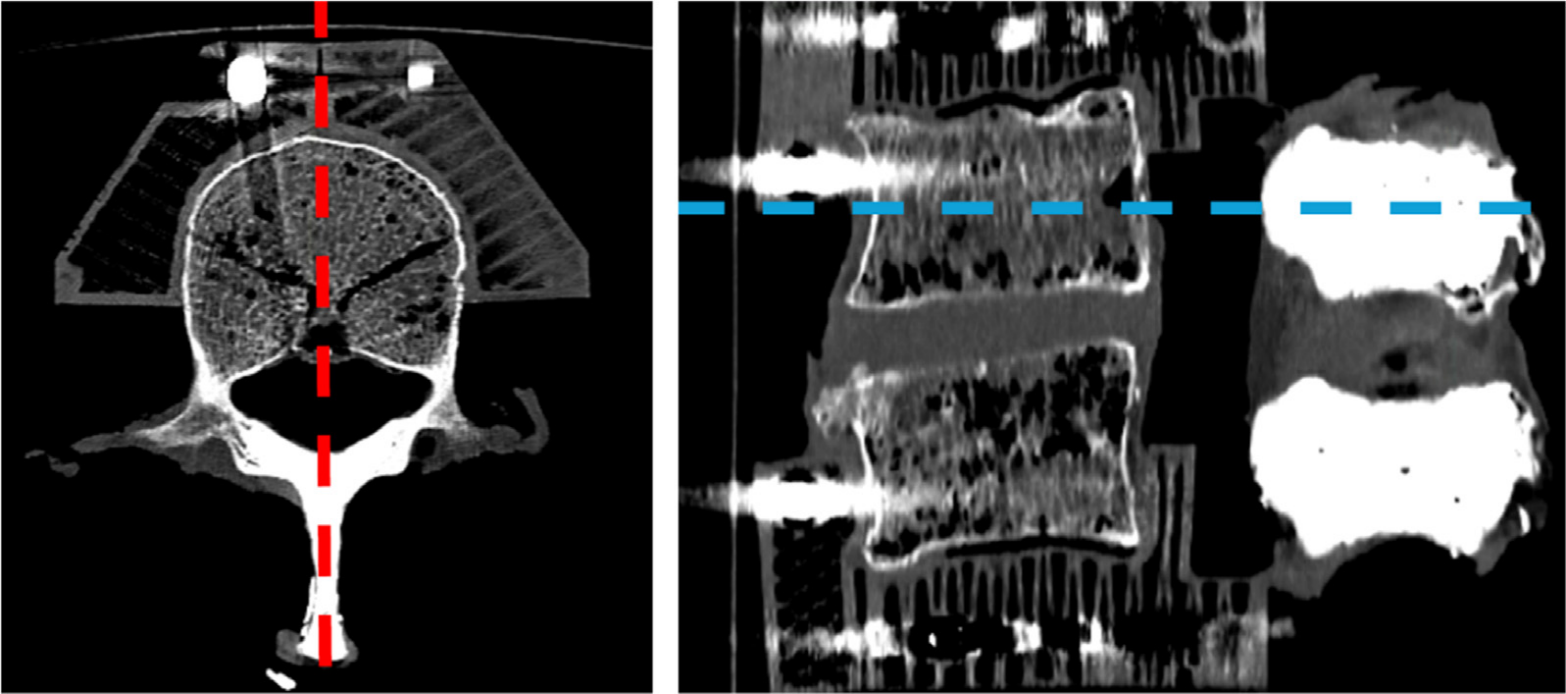
The complete augmentation of the spinous process and parts of the lamina with PMMA was clearly visible in the CT scan.

A new CT scan (Fig. 2) was performed to confirm adequate augmentation until the lamina. To emulate decompression in case of stenosis and to produce a clinically relevant weakening of the bone and of the stabilizing soft tissue, a standard midline decompression was performed on the cranial edge of the cranial SP and the caudal edge of the caudal SP using an osteotome and Kerrison punch, ensuring the facet joints remained unharmed. The remaining adjacent LF was exposed and removed cranially and caudally from the segment.

### Biomechanical testing design

Previous studies on VPX have measured the forces applied to the SPs during flexion [18]. Physiologically, spinal flexion involves the compression of the intervertebral disc's anterior portions and the anterior ligaments' relaxation relative to the center of rotation (COR). Simultaneously, the posterior structures—including the PLL, FJC, LF, ISL, and SSL—undergo stretching and experience tensile forces relative to the COR. Understandably, the previous studies overlooked the forces transmitted to the SP through the adjacent ISL, SSL, and LF. This omission occurred because the force pathways through these structures were disrupted during the preparation and isolation of each single segment.

Additionally, the remaining posterior structures did not significantly influence the flexion of the studied segment, as the tightened tendon loops diminished their restrictive effect. When these loops are tensioned, they become the primary structures restricting flexion, causing slight extension and subsequent relaxation of the ligaments and FJC. Consequently, these structures cannot resist flexion as long as the tendon loops remain intact.

To study the physiological load on the SP during flexion, it is crucial to identify the forces generated by the structures anchored to it. The tendon loops generate forces on 1 end (either upper or lower). At the same time, the other stabilizing structures contribute no significant flexion-restricting effects as long as the tendon loops remain intact, resulting in minimal contribution to the total load on the SP. On the adjacent end, forces are applied homogeneously through the posterior structures, which should remain intact.

**Fig. 3:**
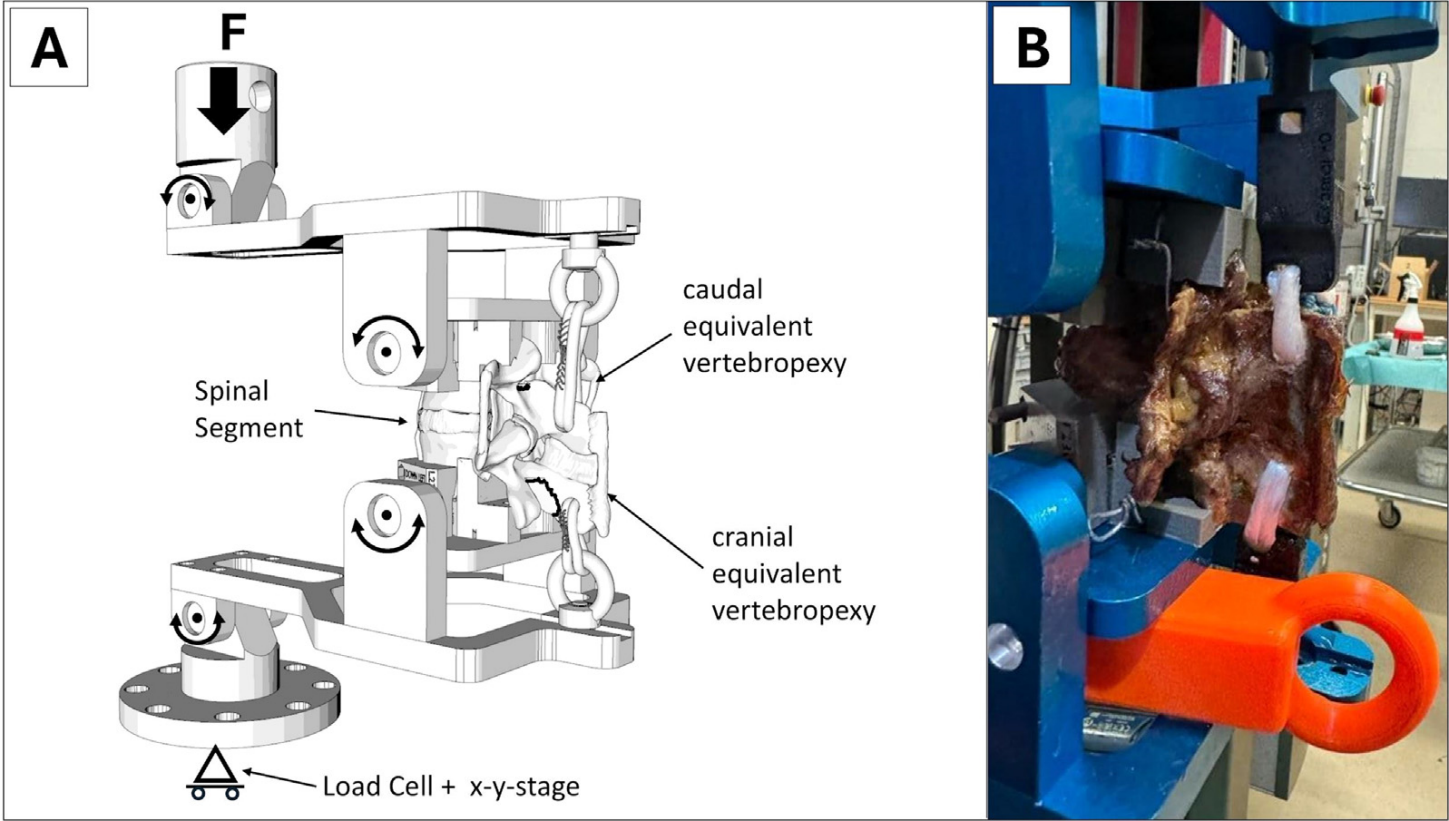
(A) The new setup to test the resistance of the process in flexion. (B) Mounting of the spinal segment on the Zwick machine.

A recently developed biomechanical setup was used to conduct the in vitro experiments [18] (Fig. 3). It adopts a reversed approach to simulate these forces by removing only the minimally contributing intersegmental structures and preserving the adjacent posterior structures. This configuration can be modified so that the upper cranial vertebra of each segment is treated as the lower caudal vertebra and vice versa.

To generate torque resulting in flexion of the spinal segment, a custom-designed, 3D-printed moving platform was utilized. This platform was engineered to produce torque through tendons anchored on the SPs by converting the compressive forces of the machine.

### Biomechanical testing protocol and experiments

The biomechanical testing of the twelve specimens was conducted using a biaxial (linear and torsion) static testing machine (Zwick/Roell Allroundline 10 kN with testXpert III Software, ZwickRoell GmbH & Co. KG, Germany), onto which the above-described setup was mounted. The interspinous Tunnel-Double Loop VPX technique was employed as previously described [21] using bovine tendons [9].

Following preparation, the segments were mounted on the testing machine using custom 3D-printed clamps [Fig. 3B].

In our experiment, the tendons were looped through the cranial SP and the socket of the moving platform and between the caudal SP and the moving platform. We used the “first-generation” VPX technique to conduct a proof-of-concept investigation under worst-case scenario conditions. Each tendon was tensioned with a force of 70 N [21] using a 3D-printed hand tightener. Two Prusik knots were stabilised on the edges of the tensioned tendon, and the free-hanging ends of the Prusik knots were subsequently tied together using a surgical 2-hand ligature knot [22]. A torque-to-failure test was conducted in 2 phases. In the first phase, torque was applied to the SP via the tendon loops, and the torque-to-failure of the entire VPX construct, defined as the fracture of an SP, was recorded. The vertebra with the fractured SP was stabilized in the second phase by fixingthe movable platform either under or over its clamp, depending on whether it was the cranial or caudal component. The experiment was then repeated to determine the resistance torque of the segment's unfractured SP.Based on the ultimate tensile strength [N], the Zwick machine forces were adjusted according to the flexion angle of the Spine Flexer and the corresponding torque values were determined by multiplying the adjusted force by the Spine Flexer’s lever arm (0.185 m).

### Data Analysis

Statistical analyses were conducted using R (version 4.3.2; R Core Team, 2023) within the RStudio integrated development environment (version 2023.09.1+494; RStudio Team, 2023). Due to the violation of normal distribution assumptions (Shapiro-Wilk test), nonparametric tests were employed. The Mann-Whitney U test was utilized to compare the Native and PMMA groups. Kruskal-Wallis tests and Bonferroni-corrected pairwise comparisons were performed to determine whether there were differences between segment-level groups. The raw failure torque and torque normalized by vertebral body bone density are reported as medians with interquartile ranges. We used Pearson correlation for normally distributed values, whereas Spearman correlation was used for non-normal distributions. We assessed the linearity using the Linear Regression Analysis and the Lack-of-fit Test.

To account for potential differences in the testing protocol caused by the stabilization of the fractured vertebra of the segment, in addition to analysing the pooled results for all SPs, we proceeded with subgroup analyses within the groups 1st and 2nd SPs to be fractured. The analysis of the 1st SPs to fracture also reflects the overall resistance of the Vertebropexy construct. This approach was designed to minimize bias and make comparisons between similarly tested SPs.

A significance level (α) 0.05 was applied for all statistical comparisons.

## Results

**Fig. 4:**
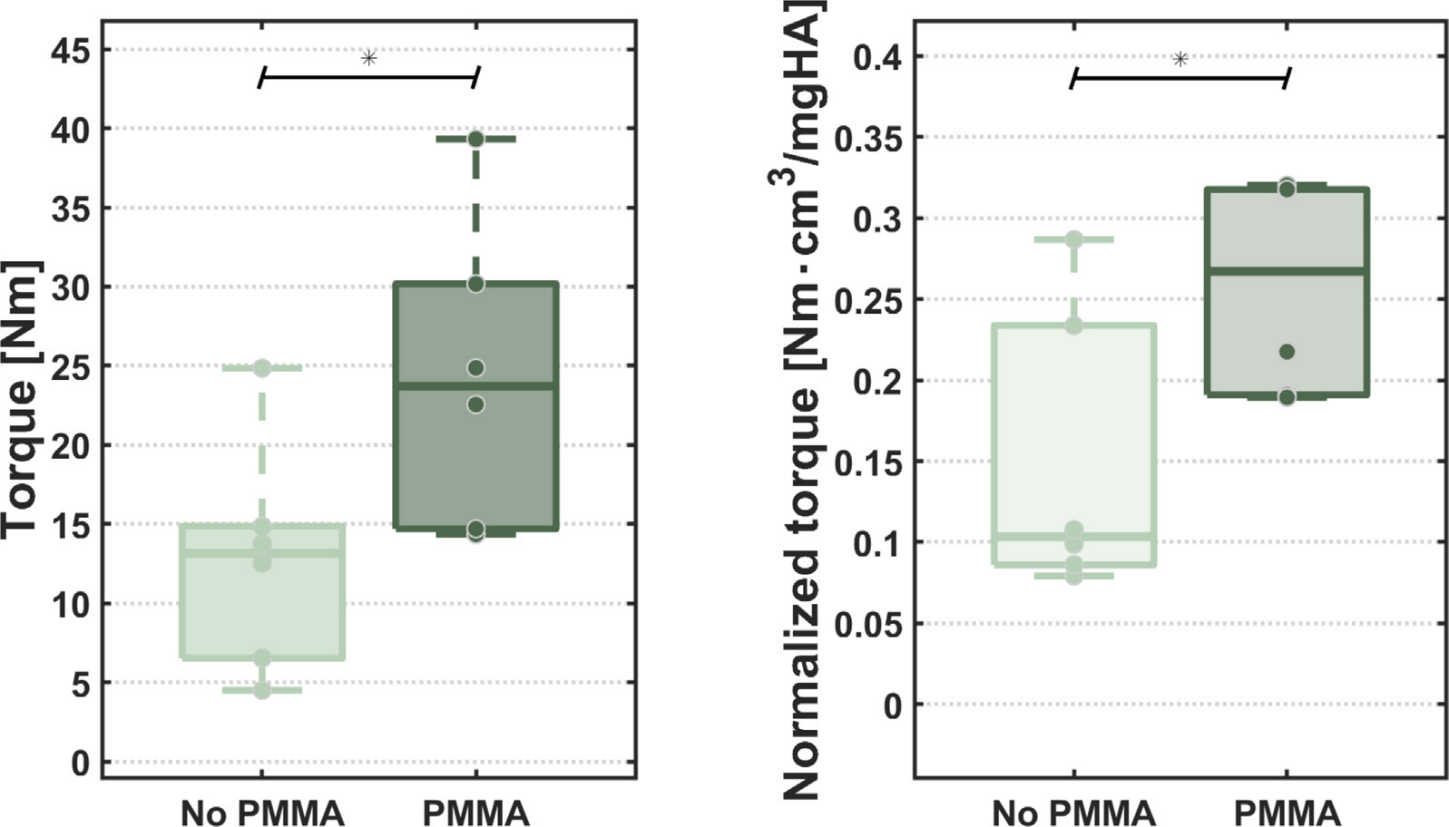
Statistically significantly higher resistance/ torque of the PMMA Group in the subgroup analysis of the first Processes to brake, which mirrors the maximum torque to failure of the whole VPX construct, resistant to normalization with the bone density of the vertebral body.

**Fig. 5:**
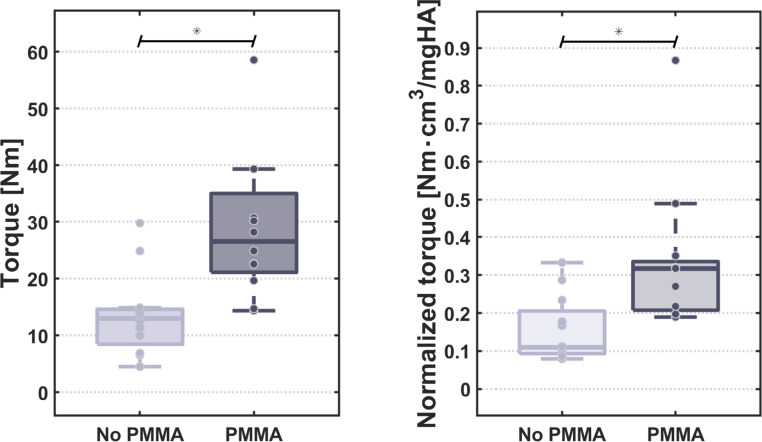
Statistically significantly higher resistance/ torque of the PMMA group, resistant to normalization with the bone density of the vertebral body.

The major finding of this study was a statistically significant difference between PMMA and Nativ group in the group with the 1st processes to be fractured which also reflects the resistance to failure of the VPX construct (p(overall) = 0.04; Median & IQR: Native 13.4 & 6.7 Nm vs. PMMA 23.7 & 19 Nm- Fig. 5). This difference was also significant in the group of the 2nd to break SPs (p(overall) = 0.01; Median & IQR: Native 12.4 & 3.2 Nm vs. PMMA 29.2 & 6.6 Nm). Additionally, the study demonstrated a statistically significant increase in resistance force/torque-to-failure for the pooled PMMA group (p(overall) = 0.00037; Median & IQR: Native 13.0 & 5.2 Nm vs. PMMA 26.5 & 11.1 Nm – Fig. 4).A normalization of torque by bone density of the vertebral body did not increase this significance (p=.001).

**Fig. 6:**
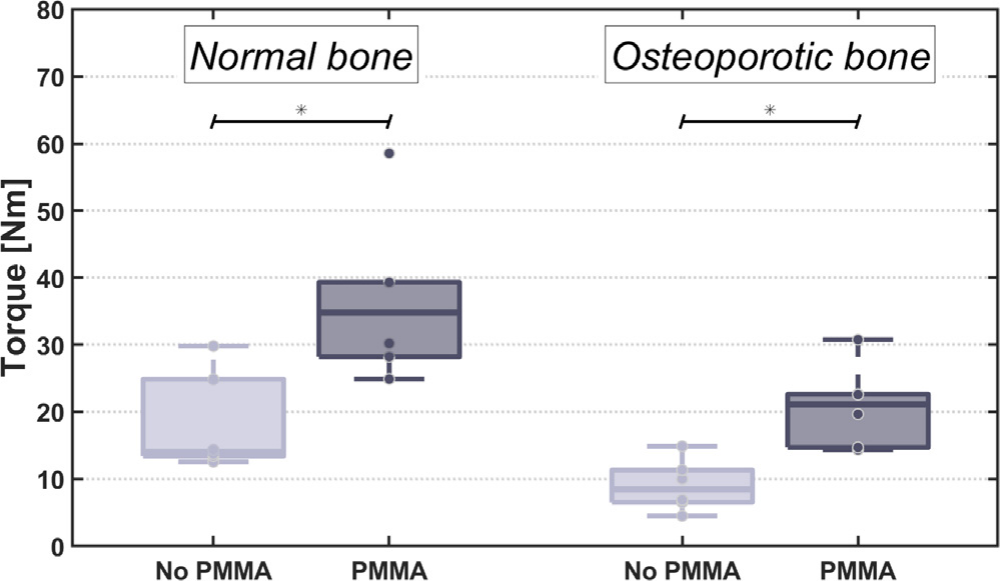
Statistically significantly higher torques of the nonosteoporotic spinous processes, both in the PMMA and Native groups and overall statistically significantly higher torques of the PMMA augmented processes in each group.

A significant difference between PMMA and Native specimens in osteoporotic and normal bone quality (p (in both) = 0.008) subgroups was found (Fig. 6). However, this difference was not significant in the analysis of the 1st (p=.1) and 2nd (p=.4) SPs to be fractured, although the medians in each analysis were clearly higher in the PMMA group (1st to be fractured: Normal 29.3 vs. 14.3 Nm; Osteoporotic 14.7 vs. 6.5 Nm). No significant difference was found when we compared the torque-to-failure of the Nativ/ NonOsteoporotic Specimens with the PMMA augmented osteoporotic segments both in the pooled results (p=.240) and in the subgroup analysis of the 1st SPs to be fractured (mirroring the construct maximum load to failure- p=.7) and the analysis of the 2nd to be fractured (p=.4).

The Nonosteoporotic specimens showed an overall statistically significant higher resistance inside the PMMA (p=.015) and Native groups (p=.025, which was not significant when tested after normalization by bone density (p=.937 and p=.699 respectively). A statistically significant linear correlation between increased bone density and bone resistance could not be detected in this cohort, which may be attributed to the small sample size. Overall: Spearman=0.276 p=.192, / PMMA: Pearson 0.474 p=.119, R² 0.225 Slope 0.181, p=.119 / Native: Pearson 0.221 p=.490, R² 0.049 Slope 0.046 *p* = 0.489).

There was no significant difference in bone density between the PMMA and Native Groups, segment-level groups, or cranial/caudal SPs. It is also important to point out that there was no statistically significant difference between torque-to-failure in general and in PMMA vs. Native Group between the cranial/caudal SPs (p(overall)= 0.477 and 0.588 (PMMA) vs. 0.393(Native)), the 1st and 2nd SPs to be fractured (p(overall)=.755 and 0.588(PMMA) vs. 1.000(Native)) and segment-level groups (T12L1 vs. L2L3 vs. L4L5- p(overall)=.761 and 0.926(PMMA) vs. 0.472(Native)). Moreover, there was also no difference in torque-to-failure, when the corresponding values were normalized by the bone density of the corresponding vertebral body. We found no significant differences between the torques to failure between PMMA or Native groups across the pooled population (containing SPs broken 1st or 2nd) and the groups of the 1st versus the 2nd SPs to fracture. This suggests that our methodology behaved consistently and homogeneously across both phases of the experiment. For this reason and because the aim of this study is primarily to show the effect of the PMMA and not to quantify the absolute loads to failure of a segment, we present only the pooled results for all fractures (i.e. the results for all the tested SPs).

**Fig. 7:**
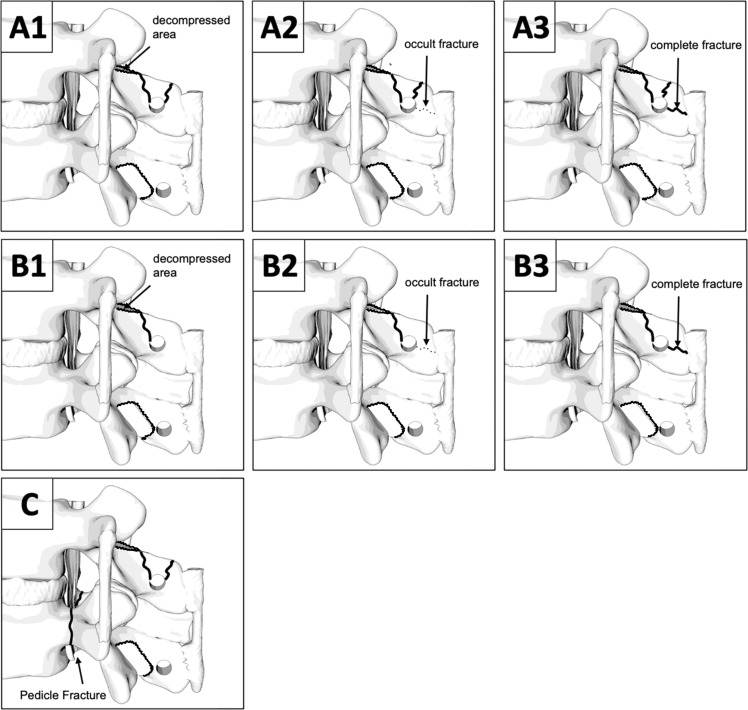
(A1) Fracture-Pull out through the decompressed parts of the spinolaminar junction (stable SSL anchoring point) (4 Fractured Spinous Processes (SPs) out of 24 SPs in total), (A2) Fracture-Pull out through the decompressed parts of the spinolaminar junction + posteriorly running occult fracture (stable SSL anchoring point) (2 Fractured SPs), (A3) Fracture-Pull out through the decompressed parts of the spinolaminar junction + posteriorly running dislocated fracture (unstable SSL anchoring point) (3 Fractured SPs), (B1) simple pull out through the decompressed parts of the spinolaminar junction (stable SSL anchoring point) (5 Fractured SPs), (B2) simple pull out through the decompressed parts of the spinolaminar junction + posteriorly running occult fracture (stable SSL anchoring point) (6 Fractured SPs), (B3) Fracture-pull out through the decompressed parts of the spinolaminar junction + posteriorly running dislocated fracture (unstable SSL anchoring point) (3 Fractured SPs), (C) Bilateral pedicle fracture - highly unstable (1 Fractured specimen).

The following modes of failure shown in Fig. 7 were identified. However, in 3 cases, there was a failure of the Prusik knot, the tendon, or a failure of the clamps. A careful examination revealed a loose knot-tendon interface, an already weakened tendon mass (due to the removal of a large amount of fibers during preparation), and a clamp with a very small clamp-bone contact area, resulting in minimal stabilization of the specimen. The bone remained intact in all cases. All cases were revised, and the tests were repeated, this time including only the load to failure of the SP in the analysis, rather than the load to failure of the entire setup. In 1 case of the augmented group, we observed a bilateral pedicle fracture of the 2nd vertebra to be fractured. In the same case, we also observed a fracture of the anterior contact point of the vertebra and the cranial clamp.

## Discussion

Semi-rigid segmental stabilization techniques (concept of VPX) can provide an alternative to spinal fusion without the extermination of the segmental mobility that results in altered biomechanics on adjacent segments [23], which is associated with high rates of revisions [6,24,25]. The mechanical resistance of the SP is crucial to the performance of these techniques [9,21,14]. Given that the total resistance is reduced after decompression [17], it is pivotal to develop a viable solution to enhance bone biomechanical characteristics. Meanwhile, several VPX technique modifications (including drilling of a much smaller hole or no drilling at all) have been described to counteract the risk of fracture of the through the SP VPX [18]. We aimed to investigate whether the enforcement of the bone with PMMA can add to the biomechanical resilience against SP fractures. For that, we used the “worst-case scenario.” of the VPX techniques, namely the first-generation VPX technique. By using the “weakest” first-generation VPX technique as a proof-of-concept model to investigate targeted improvements, the present study retains clear scientific relevance as the very “weaknesses” of the technique make it a particularly suitable reference point for comparative analysis. In this way, the observed improvements can be quantified more precisely, and their potential applicability to newly developed techniques can be discussed. However, it is possible that the findings and benefits of this method cannot be directly translated to the most modern, bone-integrity-preserving VPX techniques (eg, the Cortical Wrapping Only technique [18]). The benefit of PMMA may also be less significant when applied to these methods, as the SP's resistance may depend more on other factors, such as the quality and thickness of the cortical bone. Conversely, the “weakest” VPX technique—which preserves the ligaments of adjacent segments—could, after augmentation, become a viable alternative.

The main finding was that bone PMMA augmentation significantly increased the resistance to failure of both the whole VPX construct and the SPs separately under flexion moment, when failure was defined as fracture. Notably, the median force and torque necessary to induce a SP fracture were approximately doubled, showing also very similar resistance values (26.5 Nm) with the “safer” VPX techniques (eg with the Cortical wrapping only technique – 25.8 Nm [18]). This value is nearly half the estimated total torque exerted on the entire lumbar spine during maximum torso flexion (55 Nm in an average European weighing 70.2 kg, with a lever arm of 30 cm relative to the lumbar spine, and 69%–74% of this torque compensated by the posterior osteoligamentous complexes across all lumbar segments) [26]. This similarity suggests that PMMA augmentation could raise an outdated and risky technique to the baseline standard of current bone-preserving methods. However, no direct comparison can be made to physiological torques experienced by the lumbar VPX-stabilized SPs during everyday activities, and the answer to the question whether the augmentation can lead to fewer fractures in the patients can only be given by a clinical study.

Moreover, a subgroup analysis showed increased resistance of the augmented SPs in osteoporotic and nonosteoporotic specimen groups, as well as no significant difference between the NonAugmented/ NonOsteoporotic Specimens and the augmented osteoporotic segments. These facts highlight the method's potential not only as a protective measure in cases of osteoporosis but also as a possible refinement of the VPX technique. Our findings regarding the Native group were slightly lower than the previously published median (13.4 Nm, [18]), probably due to the inclusion of highly osteoporotic segments.

Our second finding was that in the first-generation VPX [27], a statistically significantly higher median torque-to-failure was observed in nonosteoporotic vertebrae. However, this study did not detect a statistically significant linear correlation between bone quality and maximum torque-to-failure. Although such a relationship may exist, it might not be detectable in a small sample like this. Additionally, wrapping the stabilizing material at least once around the cortical bone could further enhance the biomechanical performance of the SP [18]. Taking all the above into consideration, it can be assumed that additional factors not assessed by quantitative computed tomography of the vertebral body—such as SP cortical thickness, local bone architecture, and the relative importance of cortical bone quality compared to trabecular bone density—may play a significant role in determining the torque to failure in flexion of the SP after VPX.

Moreover, the increased resilience resulting from SP augmentation that could prevent fractures of the SPs could lead instead to the earlier onset of most clinically severe complications, like pedicle fractures, as observed in 1 specimen. This observation suggests that, while augmenting the SP and strengthening can be effective, it can also transfer failure stress to another anatomical location—such as the lamina, pars interarticularis, or pedicle—due to the over-stiffening of a structure that is typically more flexible and stress-absorbing. Although this incident occurred during biomechanical testing after excessive flexion loading, it may hold considerable clinical relevance, particularly in cases of trauma or high-impact activities that could result in acute or stress fractures. In the current study, the posterior arch was fully augmented up to the lamina. These findings suggest that the goal of augmentation should not be maximal reinforcement, but rather a risk-benefit balancing approach to prevent the creation of new failure pathways.

The study attempted to use the previously mentioned concepts for PMMA augmentation of the posterior arch [28,29]. Although the technique has only been clinically used to prevent SP fractures following interspinous spacer implantations, it seems to provide a highly resistant anchoring point for VPX and protect severely osteoporotic VPX patients. By reinforcing the SP, PMMA augmentation could provide an additional clinical strategy to reduce the risk of fractures. The findings of this study may therefore have direct implications for surgical practice in patients with reduced bone quality.

In our study, no leakage of PMMA in the spinal canal anterior to the LF occurred, even in our highly vascular osteoporotic specimens. A minimal amount of PMMA leakage (0,01 −0,02 ml) was observed posterior to the LF at its junction with the lamina in 5 SPs. This satisfactory result may be attributed to the fact that the anterior cortex of the lamina is thick and without vascular canals [30]. However, a thorough inspection for such leaks should be an essential part of the subsequent decompression process.

PMMA leakages posterior to the lamina and lateral to the SP, most likely attributable to leakage through the vascular canals in this area, were addressed by removing all leaked PMMA while it was still liquid, using our open technique under direct visualization.Pressurizing the SP from the lateral side with the surgeon's finger while injecting it with PMMA minimized the leakage. Unfortunately, it was impossible to assess the effect of leaving the paraspinal muscles anchored to the SP in reducing the leakage. This situation could simulate a case of percutaneous augmentation prior to VPX. Although such leakage may cause local tissue irritation and scarring, Bonaldi et al. [29] reported that it occurred in 2 out of 19 patients, both of whom remained asymptomatic. It is possible that the intact periosteum and paraspinal musculature play a significant role in containing the leakage.

Although the PMMA leakage with our method seems to be acceptable, it is essential to remain alert and try to prevent an anterior iatrogenic breach of the lamina, which can lead to the catastrophic event of leakage of the whole quantity of PMMA in the spinal canal. The risk may be reduced with a more appropriate diameter of a PMMA needle (13 or 14 gauge instead of 10) posterior to the spinolaminar junction. The CT-guided augmentation in a preoperative setting has also been proven clinically safe and without PMMA-related complications [31].

Moreover, in pilot tests prior to our study, we also experimented with PMMA augmentation after decompression, leading, as expected, to leakage of most of the PMMA through the decorticated parts of the bone. Therefore, augmentation should be performed before decompression. According to an older study where extraosseous leaks were observed, the surgeons stopped and reinitiated the injection after 8–10 minutes. After hardening the leaking PMMA and closing the leaking fissure, it became possible to resume the injection safely. The augmentation usually required 10–15 minutes [29].

Care must also be taken not to leave any air bubbles in the canal created by the needles, leading to areas that are not augmented.

One should also consider the risk of excessive hardening of the SP or lamina due to PMMA, which can cause difficulties during decompression (with burr, Kerrison punch, or chisel) either during primary VPX or later in revision surgeries. Accidental augmentation of the pedicles may also lead to screw deviations (in cases of partial augmentation in small pedicular areas) or even make pedicle screw placement impossible (if a PMMA bolus is present in the middle of the pedicle). It is recommended to limit augmentation to the maximal spinolaminar junction under continuous fluoroscopic or CT guidance to minimize such complications.

The effects of the PMMA on the bone biology of the SP and the long-term outcomes of the method, for example, through thermal necrosis due to the exothermal polymerization reaction, cannot be predicted [32] Temperatures at the bone–cement interface can reach 67°C to 124°C, with thicker cement mantles producing higher temperatures and, consequently, more necrosis. The exposed ends of the trabeculae, embedded in the cement, are most susceptible. The threshold for immediate thermal necrosis of osteocytes has shown considerable variability in the literature [[33], [34], [35], [36], [37], [38], [39]]. It is primarily based on rabbit studies, with 30 seconds at 50°C [40] or 1 minute at 47°C [41] being the most widely accepted limits. Additionally, exposure to temperatures above 45°C for 15 seconds has been shown to cause osteocyte damage [42]. The relationship between temperature and exposure duration is logarithmic: a slight increase in temperature can significantly reduce the time required for necrosis [43]. The impact of thermal necrosis on tendon-to-bone healing has not yet been studied, but it likely leaves a zone of damaged cortical bone with minimal or no healing potential. Additionally, drilling through PMMA-augmented bone—which is more challenging and time-consuming—generates further heat, potentially exacerbating thermal injury. The affected bone biology could also result in altered tendon tissue integration/tendon-to-bone healing. However, it is possible that bone remodelling due to altered load balance after VPX can cause a late failure of the construct. Further, because standard PMMA is biologically less active [36,37,44], integration cannot be guaranteed in regions where the tendon comes into contact with PMMA, particularly in the tunnels. This poses a significant risk, as the long-term goal of VPX (tendon-to-bone healing) might be compromised. However, there are currently several promising strategies, which could become an almost mandatory part of PMMA procedures in the future, such as precooling [45], as used in our study and mixing PMMA with bioactive agents [46,47] to enhance osteoconductivity and mechanical properties, and most importantly, to reduce the peak temperature of exothermic polymerization [48]. This could lead to temperatures that not only limit thermal necrosis, but also potentially enhance bone regeneration via localized thermal necrosis [37,49]. In a clinical study investigating the outcome of patients after SP augmentation with PMMA before insertion of an interspinous spacer, no remodelling of the posterior arch was observed 1 year postoperatively [29].

Potentially, a weakening gigli-saw effect of the tendon on the bone, as well as all the complex and multidirectional forces, were not encompassed in the study, and the role of paraspinal muscles could not be assessed. However, the study evaluated the mechanics of the SP fracture due to bending moments, which appears to be the most significant reason for failure [18,50].

Furthermore, increasing the elastic moduli of the bony structures with PMMA can alter the strain distribution in the vertebra, leading to new types of clinical failure and possibly catastrophic events such as a bilateral pedicle or laminar fracture, which was seen in 2 of our specimens. Exaggerating with PMMA augmentation could also lead to a filling of the pedicle medullary bone with PMMA, with subsequent challenges in case of a spinal fusion. In the previous studies, 1–2 mL of PMMA was injected without complications [29,31].

Bovine tendons were used instead of allografts, as their stiffness and failure loads are similar to human cadaveric tendons in in vitro studies [51].

The final limitation of this study, alongside the small sample size, is the analysis of pooled experimental results (torques to failure) from both the 1st and 2nd SPs to fail. After the stabilization of the 1st vertebra to fail, the center of rotation shifted anteriorly, increasing the lever arm and consequently reducing the force/torque needed to cause failure in the 2nd SP. Including these reduced torques in the analysis may decrease the reported overall torque to failure. However, this compromise was accepted because it does not overestimate the torque to failure, which could entail associated clinical risks. Moreover, the goal of the study was not compromised, as the aim was to demonstrate an increase in load to failure, rather than to determine the exact load to failure in flexion. Even with this approach, the results remained statistically significant.

Despite the current uncertainties regarding the optimal quantity of PMMA to balance mechanical strength, leakage control, and bone biology, PMMA augmentation of the SP demonstrates considerable potential as a valuable and impactful tool in clinical practice.

## Conclusion

The PMMA-augmentation of the spinous processes can multiply the torque-to-failure/fracture in osteoporotic and nonosteoporotic conditions when performing Verterbropexy. Nonosteoporotic specimens show a statistically significant higher resistance than osteoporotic specimens, both with and without PMMA augmentation. This study proposes a cement augmentation technique of the spinous process to minimize failure risk in the setting of first-generation through-the-spinous-process Vertebropexy.

## Supplements

### Supplementary Information

A supplement with the descriptive statistics and statistical tests can be added when needed.

## Declaration of generative AI and AI-assisted technologies in the writing process

During the preparation of this work, the author(s) used Grammarly for language correction. After using these tools, the author(s) reviewed and edited the content as needed and take(s) full responsibility for the content of the publication.

## Funding

This study did not receive any specific grant from funding agencies in the public, commercial, or not-for-profit sectors. The work was supported solely through internal departmental funding at Balgrist University Hospital.

## Ethics approval

Kantonale Ethikkommission Zürich had given the approval for the study. (Basec No. KEK-ZH—Nr. 2022-00715).

## Author contribution

***AT:*** Conceptualization, Data curation, Formal analysis, Investigation, Methodology, Project administration, Resources, Writing – original draft, Writing – review & editing. ***MRF:*** Data curation, Formal analysis, Methodology, Writing – review & editing. ***OW:*** Data curation, Investigation, Methodology, Software. ***MF:*** Conceptualization, Funding acquisition, Methodology, Supervision, Validation, Writing – review & editing. ***JW:*** Conceptualization, Formal analysis, Investigation, Methodology, Project administration, Resources, Supervision, Writing – review & editing.

## Declarations of competing interests

One or more of the authors declare financial or professional relationships on ICMJE-NASSJ disclosure forms.
